# Increased Vaccination Diversity Leads to Higher and Less-Variable Neutralization of TBE Viruses of the European Subtype

**DOI:** 10.3390/vaccines11061044

**Published:** 2023-05-31

**Authors:** Malena Bestehorn-Willmann, Philipp Girl, Franziska Greiner, Ute Mackenstedt, Gerhard Dobler, Daniel Lang

**Affiliations:** 1 Institute for Zoology, Parasitology Unit, University of Hohenheim, 70599 Stuttgart, Germany; malena.bestehorn@googlemail.com (M.B.-W.); franziska.greiner@uni-hohenheim.de (F.G.); mackenst@uni-hohenheim.de (U.M.); 2 Bundeswehr Institute of Microbiology, 80937 Munich, Germany; philipp.girl@web.de (P.G.); daniellang@bundeswehr.org (D.L.)

**Keywords:** tick-borne encephalitis virus, European subtype, vaccination-induced antibodies, microtiter-neutralization assay

## Abstract

Tick-borne encephalitis (TBE) is an infectious disease of the central nervous system. The causative agent is the tick-borne encephalitis virus (TBEV), which is most commonly transmitted by tick bites, but which may also be transmitted through the consumption of raw dairy products or, in rare instances, via infected transfusions, transplants, or the slaughter of infected animals. The only effective preventive option is active immunization. Currently, two vaccines are available in Europe—Encepur^®^ and FSME-IMMUN^®^. In Central, Eastern, and Northern Europe, isolated TBEV genotypes belong mainly to the European subtype (TBEV-EU). In this study, we investigated the ability of these two vaccines to induce neutralizing antibodies against a panel of diverse natural TBEV-EU isolates from TBE-endemic areas in southern Germany and in regions of neighboring countries. Sera of 33 donors vaccinated with either FSME-IMMUN^®^, Encepur^®^, or a mixture of both were tested against 16 TBEV-EU strains. Phylogenetic analysis of the TBEV-EU genomes revealed substantial genetic diversity and ancestry of the identified 13 genotypic clades. Although all sera were able to neutralize the TBEV-EU strains, there were significant differences among the various vaccination groups. The neutralization assays revealed that the vaccination using the two different vaccine brands significantly increased neutralization titers, decreased intra-serum variance, and reduced the inter-virus variation.

## 1. Introduction

Tick-borne encephalitis (TBE) is a severe, vector-borne disease that affects the central nervous system. The infectious disease is common in parts of Europe and Asia. In Europe, it has been a notifiable disease since 2012 and has become a growing public health challenge, with distinct regional epidemiology. Countries such as Sweden and Finland display continuous increases in TBE case numbers. In Germany, Switzerland, France, the Czech Republic, and the Slovak Republic, a pronounced increasing trend in TBE case numbers has been recognized since 2016. Strikingly, even in Austria, with high vaccination rate of 85%, this clearly rising trend in TBE cases is recognizable [1]. In addition, TBE has occurred in regions that have been considered, so far, to be TBE-free [2,3]. 

Additional potential risk areas could be identified by seroprevalence studies of sentinels [4,5]. The causative agent, the tick-borne encephalitis virus (TBEV), a positive-strand RNA virus from the family *Flaviviridae*, is transmitted to humans mainly by the bite of an infected tick. In Central Europe, primarily hard ticks of the species *Ixodes ricinus* function as vectors. In other parts of Europe and Asia, *Ixodes persulcatus* (taiga tick), *Dermacentor reticulatus*, *Ixodes ovatus*, or *Ixodes nipponensis* may play a role. Furthermore, TBE infections may be acquired by the consumption of raw dairy products [6,7,8] and possibly by organ transplants or blood transfusions of patients in the viremic phase, by breast-feeding from acutely infected mothers, by aerosol (laboratory infections), or by direct contact with the viremic blood of animals (e.g., slaughtering). Phylogenetically, TBEV can be divided into three to five major subtypes [9,10,11] that have different geographic distributions and manifest in different forms of the disease, with varying severity [12,13,14,15,16,17]. TBEV-EU represents the prevalent subtype and evolutionary TBEV lineage in Europe, possibly tracing to early 1900 and expanding in effective population sizes from east to west until the 1970s. Extant TBEV-EU genotypes persist endemically in local microfoci that can be traced to specific, older phylogenetic clades and, presumably, to long-range dispersal by random chance encounters with migratory animals and humans [18,19].

To prevent the disease, active immunization against TBEV with an inactivated vaccine is recommended by the WHO and also by various national health agencies [20]. Two vaccines are available in Europe, which are based on two distinct TBEV-EU subtype virus strains. Both vaccines contain formalin-inactivated virus particles and are produced in primary chicken fibroblast cell culture. The vaccine Encepur^®^ (Bavarian Nordic GmbH, Planegg, Germany) is based on the TBEV strain *K23*, which was originally isolated in the area of Karlsruhe, Germany in 1975 [21]. The vaccine FSME-IMMUN^®^ (Pfizer, Vienna, Austria) is based on the strain *Neudörfl* that was isolated in the eastern lowlands of Austria in 1971 [22,23].

Even small amino acid changes in specific positions of the TBEV envelope (*E*) protein may alter relevant epitopes of the antigen and, thus, the affinity and specificity of the antigen–antibody complex. The two strains that were used to generate the European vaccines differ in 32 amino acids on the whole polyprotein and in four amino acids on the envelope protein scale. So far, it is unclear whether antibodies induced by the vaccine-contained virus strains produce antibodies that react against all naturally circulating virus strains from TBEV-EU. Previous experiments involving micro-neutralization test (NT) assays compared strains from different subtypes or different flaviviruses with each other [21,24,25,26,27]. These studies showed positive neutralization results among different subtypes and even among flaviviruses from different sero-complexes. Most of these experiments were carried out with established reference strains that are often used as vaccine strains, with have a long cultivation history in cell-based systems. Consequently, there is a possible accumulation of mutations within those viruses because of the adaptation to the respective cell culture system [28]. There is new evidence that a single artificial amino acid substitution at residue 52 in the seed virus of the Encepur^®^ vaccine might have altered the antigenic structure of the *E* protein with a significant impact on the neutralizing capacity [29]. Therefore, to date, it has not been shown whether vaccine-induced antibodies against specific TBEV-EU strains neutralize all circulating wild genotype lineages of the TBEV-EU subtype.

As a member of the family *Flaviviridae*, TBEV exhibits ample cross-reactions with other flaviviruses in conventional IgG tests (ELISA; [30]). These cross-reacting antibodies, although not protective, might, however, interfere with the formation of neutralizing antibodies after vaccination with a non-live vaccine [31]. It is well known that the neutralization test is highly specific and does not exhibit cross-reactivity against different flaviviruses. Furthermore, in contrast to ELISA, which detects only structural classes of antibodies (e.g., IgG) the neutralization test indicates the presence of functional (neutralizing) antibodies, which are thought to play a major role in protection.

**Figure 1:**
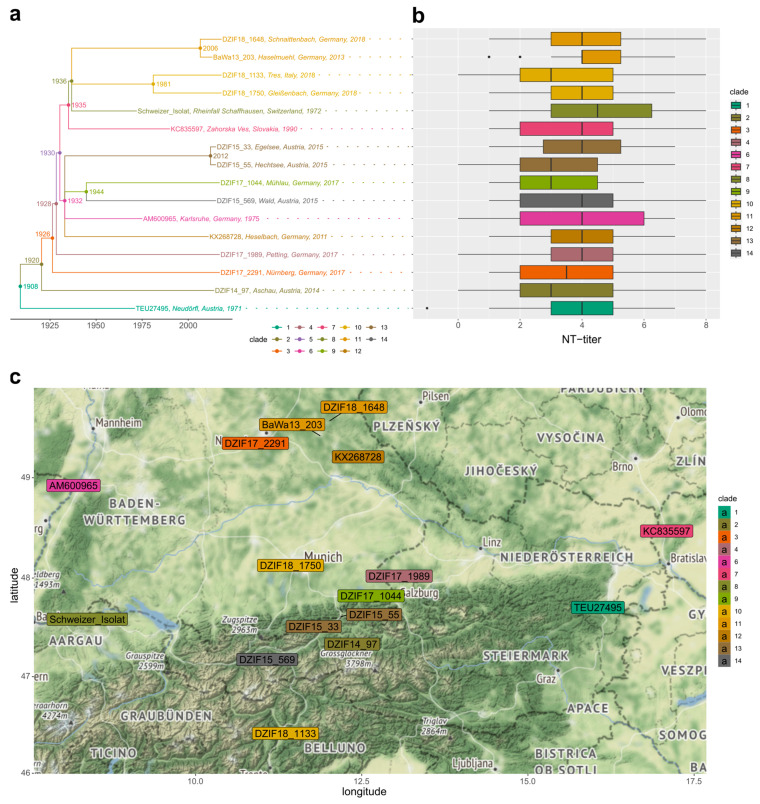
(**a**) Dated phylogenetic tree of the analyzed TBEV-EU genotypes. Internal node labels represent the inferred year w.r.t. the minimal dates of the last common ancestors. X-axis and branch-lengths are scaled according to estimated divergence dates (i.e., chronogram). Multifurcations in the depicted chronogram represent clades with bootstrap support <70% in the originating maximum-likelihood tree topology. Lineages of closely related TBEV-EU genotypes were defined by selecting groups of tips whose last common ancestor was inferred to be earlier than 1960. These phylogenetic clades (1–14) are color-coded with the same color in the tree (**a**), in the boxplot (**b**), and on the geographic map (**c**). Clade clusters (1–14) were inferred automatically. Clade 5 comprises solely an internal node and does not contain extant taxa. Clade cluster numbering was kept for consistency. (**b**) Box-whisker plot of TBEV-EU neutralization titers (NTs) by virus genotype. (**c**) Geographic distribution of the 16 TBEV-EU virus isolates used in this study. Colored text boxes depict inferred geolocations of the originating TBEV isolates. To prevent overlaps, positioning of text boxes is shifted in case of closely located isolates. A black connecting line indicates the true position of the respective geolocation in these cases.

The aim of this study was to perform NT assays comparing the neutralizing capacity of sera obtained from vaccinated persons against virus strains from different genetic clades of the European TBEV subtype. The novel virus strains were directly isolated from tick homogenates and were cultured in only one passage in cell culture before the neutralization assays to keep them as close to their genetic wild-type form as possible. To establish a more complete picture of the circulating TBEV-EU genotype lineages, we used available virus isolates from Central European TBEV hotspot regions in southern Germany, as well as from neighboring regions in Austria and Switzerland (Figure 1c).

Sixteen TBEV-EU virus strains were tested by a micro-neutralization test against a panel of 33 sera from healthy donors who had a complete basic vaccination against TBEV, according to the recommended vaccination schedules of the manufacturer. The pool of samples consisted of equal numbers of sera from persons vaccinated with FSME-IMMUN^®^, Encepur^®^, or both vaccines.

## 2. Materials and Methods

### 2.1. Human Sera

For this study, 33 serum samples from vaccinated individuals were obtained from the National Consultant Laboratory for TBEV (Bundeswehr Institute of Microbiology, Munich, Germany). We selected anonymized sera with available information on vaccination brand, the number of TBEV vaccinations, demographic data (age and sex), and additional flavivirus vaccinations (yellow fever vaccination) of the vaccinees. Testing for other flavivirus IgG antibodies (dengue virus, Japanese encephalitis virus, West Nile virus, Usutu virus) did not reveal any serological evidence of other flavivirus infection. The sample metadata are presented in Table 1.

Table 1 shows the metadata of the human sera samples used in this study. The number of vaccination shots administered, as well as the information on vaccination brands, were registered. The age of the vaccinees is given in decades of age groups. The sex of the patients and the presence of yellow fever vaccination are also specified.

### 2.2. Virus Strains

TBEV-EU genotypes were selected from the strain collection from the National Consulting Laboratory for TBEV, according to geographical origin and genetic variability. Due to a phylogenetic tree based on the open-reading frame of all available whole genome sequences (NCBI Genbank), available strains from different clades were selected. Further strains accumulating non-synonymous and unique amino acid substitutions were included in the analysis.

Sixteen TBEV strains from the European subtype were used. Twelve of the genotypes were isolated at the Bundeswehr Institute of Microbiology after 2011. These isolates were cultivated from the original tick homogenate during this study and sub-cultivated once to generate virus stock solutions for the experiments. The strains *K2* (Karlsruhe) and *Schweizer Isolat 40* and were kindly provided by F. X. Heinz (Vienna, Austria). These strains were sub-cultivated once to reassure adaptation to the A549 cell line and to evaluate the virus titer. The passage history of the remaining strains is provided in Table 2. Ten virus strains were originally isolated in Germany; 3 virus strains originated from Austria; 1 came from Slovakia; and one each came from Italy and Switzerland. Unfortunately, Baltic strains could not be included in the study, as no TBE-EU strain from the Baltic region was available during the experimental phase of the study. The proprietary vaccine strain *K23* could not be included in the experiments, but it was used for the phylogenetic analyses. Instead, the parent strain of *K23*, TBEV-EU strain *K2*, was included in the study (both strains differ in a single substitution). Two of the strains (*DZIF18_1648* and *DZIF18_1750*) were isolated from ticks in areas where infections resulting in vaccine failures occurred.

Area, year of isolation, and isolation source provided in Table 2. References were included if available. When two countries were indicated, the ticks were collected in the first-listed country, close to the border of the second-listed country. In addition, the method of sequencing and NCBI Genbank accession numbers (Acc.) of previously published strains are provided.

### 2.3. Virus Cultivation Quantification and Microserum Neutralization Tests

Virus isolation and cultivation were performed using A549 (CCL-185^™^) cells in culture medium (minimal essential medium (MEM) plus 1x non-essential amino acids (NEAA), 1x antibiotics and antimycotics (ABAM), 2% fetal bovine serum (FBS); all cell culture reagents were from Invitrogen™, Thermo Fisher Scientific, Darmstadt, Germany). Harvested cell culture supernatant containing a virus was replenished with 20% FBS and stored at −80 °C until use.

All virus strains used in this study were sub-passaged once in A549 cells. Subsequent experiments were conducted with aliquots from this sub-cultivation. The virus titers of the aliquots were determined via tissue culture-infectious dose titration in 96-well plates. Fifty µL/well of 10 log dilutions (10^−1^ to 10^−12^) in culture medium of the respective TBEV strain were pipetted. To each well, 10^4^ A549 cells in 50 µL cultivation medium were added. The 96-well plates were incubated at 37 °C and 5% CO_2_. On day 5, the supernatants were removed and 200 µL of the staining solution (10% formaldehyde, 0.1% crystal violet) was added to each well. After 30 min, the staining solution was removed and the wells were washed with tap water. The plates were dried and visually examined. Calculations of the tissue culture infectious doses (TCID) were performed according to Bear et al. [34].

Neutralization tests were performed according to standard procedures as microneutralization assays in 96-well plates, as described earlier [35]. Briefly, the serum samples were inactivated at 56 °C for 30 min. A serial two-fold dilution of the sera was made with MEM (Gibco™, Thermo Fisher Scientific, Inc.), 1× NEAA, and 1× ABAM generating dilutions from 1:10 to 1:1280. For each dilution, 50 µL were then mixed with the same amount of a virus dilution resulting in 50–100 TCID per well. Each dilution was performed in triplicate. The serum–virus mixture was incubated for 1 h at 37 °C. Then, 50 µL of A549 cells (10^4^ cells per well) were added to each well and the assays were incubated for 6 days at 37 °C and 5% CO_2_. After 6 days of incubation, the supernatant was carefully discarded and the wells were stained with 100 µL of crystal violet (10% formaldehyde, 0.1% crystal violet). The stained assays were incubated at 4 °C for at least 30 min, the stain solution was removed, and the wells were washed with tap water. The assays were then dried and examined visually. A blue ground indicated neutralization, while a translucent ground was an indication of virus growth and, therefore, no neutralization.

### 2.4. Phylogenetic Analysis of the TBEV-EU Genome Sequences

The TBEV strains that were isolated at the Bundeswehr Institute of Microbiology were sequenced using the nucleic acids that had previously been extracted from the tick homogenates. The virus strains provided by Professor Heinz (Institute of Virology, Vienna, Austria) were sequenced directly from the nucleotide extraction of the baby mouse passage provided. Subsequently, three overlapping DNA amplicons of 5.2–5.6 kb, covering the whole TBEV genome, were generated using the SuperScript™ III One-Step RT-PCR System with Platinum™ Taq High Fidelity DNA Polymerase (Invitrogen, Thermo Fisher Scientific, Darmstadt, Germany). The cDNA amplicons were mechanically sheared using a Bioruptor UCD-200 in order to obtain DNA fragments of roughly 600 bp. Library preparation was performed using the TruSeq Nano DNA Low Throughput Library Prep Kit (Illumina GmbH, Munich, Germany). The Illumina MiSeq platform was used for sequencing with the MiSeq reagent kit V3 2 × 300 (Illumina GmbH, Munich, Germany), as described by the manufacturer. De novo assembly of the raw reads into a single scaffold was performed using the software Spades v.3.12 [36]. As no RACE PCR was performed, the primer sequences were trimmed off for further analysis. In order to identify amino acid substitutions, the single open-reading frame was extracted and translated into the amino acid sequence for each sequence. Basic sequence analysis was performed using Geneious prime (https://www.geneious.com/prime/, accessed on 6 May 2023).

To assess the genetic diversity of the selected TBEV-EU isolates and to identify common phylogenetic lineages and genotypic clades, we performed phylogenetic inference based on the whole genome sequences. Metadata for all 16 TBEV-EU genotypes were collected and manually curated, with special focus on the collection dates and geolocations.

A multiple whole-genome alignment of the 16 TBEV-EU strains was calculated using MAFFT (parameters: --auto --6merpair, [37]). The multiple-sequence alignment (MSA) was utilized to select an evolutionary model, using the ModelTest-NG software [38]. The best model (GTR + I + G4) was employed for maximum-likelihood (ML) tree inference with 200 bootstrap replicates, using the RAxML-NG software [39]. In the resulting phylogenetic tree, bifurcating clades with bootstrap support <70 were collapsed into multifurcations (bML tree). The collection dates, the MSA, and the established bML tree topology were used for maximum-likelihood dating and ancestral-state reconstruction, using the coalescent and relaxed molecular clock approaches implemented in the TreeTime software [40]. To define dated phylogenetic clades and to infer divergence estimates of the last common ancestors (Figure 1a), molecular date estimates and confidence intervals were analyzed along the dated and the bML tree topologies, using the R packages tidytree, treeio, ape, phytools and ggtree [41,42,43,44,45]. Color-coded clades (Figure 1) were defined by clustering the projected dendrogram of the dated bML topology, using the ape cutree method, at height 0.1 that represented TBEV-EU lineages that diverged before 1960.

The geographic distribution of the reported geolocations from the original isolates of the selected TBEV-EU strains was analyzed and plotted with OpenStreetMap data and terrain map tiles by Stamen Design at zoom level 7, using the R packages osmdata and ggmap [46,47].

### 2.5. Statistical Analysis

All patient and serum-derived data processing, data analysis, descriptive statistics, and hypothesis-testing steps were implemented in R language and environment for statistical computing (version ≥3.6.3; https://www.r-project.org, accessed on 6 May 2023). Positive neutralization titers were scaled by dividing them by 10 and by subsequent log transformation. Negative neutralization tests (i.e., missing data) were included as −1. Normal distribution was analyzed with quantile–quantile plots, using the R qqplot method (not shown). Variable-rank correlation analysis of different data groups was performed using the Spearman’s rank correlation test. Non-parametric one-way analysis of variance between groups was assessed using the Kruskal–Wallis test. The Fligner–Killeen test for the homogeneity of NT-titer variances between immunization profiles was carried out using the R function fligner.test. The phylogenetic signal of median NT-titers for each TBEV-EU strain was tested using the Blomberg’s K method [48], with 1000 randomizations implemented in the R function phylosig from the ape package. The phylogenetic MANOVA of median NT-titers among the different phylogenetic clades across the three vaccination profiles was assessed using the Wilks test statistic with the aov.phylo function implemented in the geiger R package [49].

## 3. Results

In order to investigate whether all European TBEV strains are similarly recognized and neutralized by the available European vaccines, we used 33 serum samples from vaccinated individuals and a variety of 16 TBEV strains of the European subtype. For each serum, neutralization titers were generated using all 16 virus strains selected for the study. In 12 cases, the serum did not suffice for all virus strains. In total, 528 individual neutralization titers were generated. With the exception of one “FSME-IMMUN^®^” sample, all serum–virus combinations resulted in a neutralization titer of ≥10. The data were scaled by log_2_ (titer × 0.1) in order to eliminate the influence of the two-fold dilutions. Using this transformation, the results could be analyzed in a discrete order, simplifying the interpretation. The median neutralization titer was 4 (1:160) and the mean titer was 3.915 (1:150) The highest detected neutralization titer was 8 (1:2560) and the lowest neutralization titer was 0 (1:10).

### 3.1. High Inter-Serum Variation and Low Sampling Bias

In total, 33 sera with a minimum of three vaccinations were used in the study (Table 3). The number of vaccination shots administered ranged from three to seven, while most candidates were vaccinated five times. There was a weak positive correlation between the neutralization titer and the number of vaccination shots (Kendall’s rank, R = 0.109, *p* = 0.0018) and a significant positive impact (GLM coefficient: 0.23933, *p* = 0.00212). Female donors contributed 67% (*n* = 22) of the samples. There was no significant difference in neutralization titers between the sexes of the sample donors (Wilcoxon, *p* = 0.063). A yellow fever vaccination was administered to 27% (*n* = 9) of the donors. We tested for possible impact of a prior yellow fever vaccination and found no influence on the neutralization titers (Wilcoxon, *p* = 0.79) and very weak, but significant, negative correlation (polyserial correlation coefficient −0.0517740910; *p* = 6.391799 × 10^−17^). Unfortunately, no data on the time period between the last vaccination dose and the sampling time point were available. The samples included individuals between 20 and 60 years of age. A tendency toward higher NT-titers in middle-aged individuals ([age category] = median NT-titers: 20–30 = 3; 31–40 = 5; 41–60 = 3; Kruskal–Wallis, *p* = 8.541 × 10^−14^) was detected. Nevertheless, the uneven representation of the three different immunization profiles (see Section 3.3) among these age categories suggested caution in over-interpreting this result, and may have hinted at a pronounced underlying effect of this variable.

Overall, we detected a high inter-sera variation (Kruskal–Wallis, *p* < 2.2 × 10^−16^) coupled with a low intra-serum variance.

### 3.2. Genotypes of the Diverse and Highly Divergent TBEV-EU Panel Were Neutralized Independent of Their Phylogenetic Position

The 16 selected TBEV-EU strains were selected to capture the diversity of Central European TBEV hotspot regions in southern Germany, as well as neighboring regions of Austria and Switzerland (Figure 1c). Information about the strains’ isolation sources and origins is provided in Table 2. Figure 1 depicts the inferred phylogenetic relationships, the estimated diversification times, and the geographic distribution of the selected genotypes. Given our molecular clock estimates, the chosen 16 TBEV-EU strains fell into 13 genotypic clades that diverged sometime before 1960 (Figure 1a). According to this criterion, almost all isolates represented individual phylogenetic lineages that were at least 71 years old (i.e., the last common ancestor (LCA) of *DZIF15_569* and *DZIF17_1044* was estimated to have existed in 1944). Only clades 10 (LCA 1981; *DZIF18_1750* and *DZIF18_1133*), 11 (LCA 2006; *BaWa13_203* and *DZIF18_1648*), and 13 (LCA 2012; *DZIF15_55* and *DZIF15_33*) harbored two members each, with younger divergence time estimates.

At a nucleotide level, the viruses showed differences between one (*DZIF15_33* and *DZIF15_55*) and 530 nucleotide substitutions (*K2* and *Neudörfl*), resulting in amino acid substitutions ranging from one (*DZIF15_33* and *DZIF15_55*) to 40 (*K2* and *DZIF18 1989*). Regarding the major antigen, the *Envelope* protein, our dataset included four strains with identical amino acid sequences and 12 strains with unique amino acid sequences. The maximum difference was five amino acids (*DZIF14_97* to *DZIF17_2291*, *DZIF17_1989*, *K2* to *DZIF18_1750*).

When we tested NT-titer phenotypes among the different virus genotypes and tested for inter-strain variation, no significant differences between the viruses could be observed (Kruskal–Wallis, *p* = 0.35; Figure 1b). The variance of the NT-titers for individual virus strains was broader than it was for individual serum samples. Furthermore, there was no significant phylogenetic signal of NT-titers that were detectable along the tree topology (Blomberg’s K 0.1912986, *p*-value 0.803), as well as no significant variation of NT-titers in individual genotypic clades (phylogenetic MANOVA; Wilks test statistic = 2.6145 × 10^−5^, *p* = 0.92). In fact, all clades with more than one member (clades 10, 11, and 13) displayed highly divergent NT-titer phenotypes.

### 3.3. Neutralization Titers Differed Significantly, Depending on Serum Immunization Profiles

**Figure 2:**
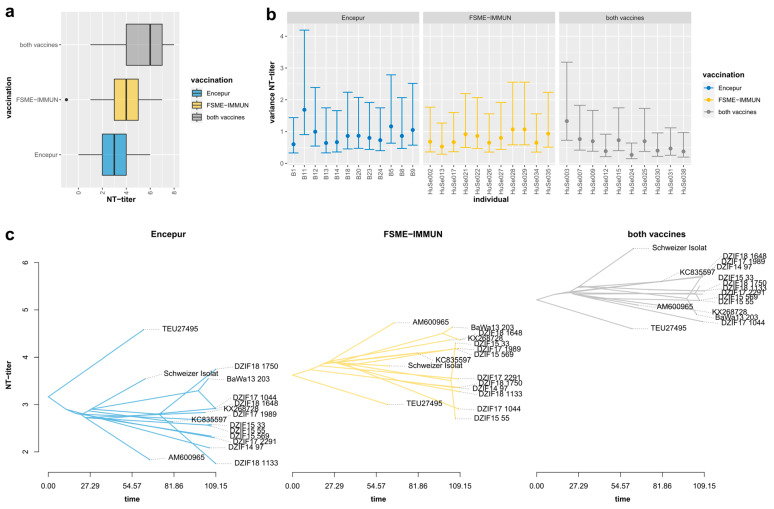
(**a**) Box-whisker plots of NT-titers grouped and color-coded by immunization profile. (**b**) Intra-serum variances of NT-titers grouped and color-coded by immunization profile. Dots represent individual serum variances. Error bars depict 95% confidence intervals of the intra-serum variance. (**c**) Phylogenetic phenogram of mean NT-titers for each TBEV-EU genotype grouped and color-coded by immunization profile. For each immunization profile, phylogenetic comparative plots are shown that project the underlying phylogenetic tree in a space defined by phenotype (NT-titer; y-axis) and time (divergence time estimates; x-axis).

In order to assess the impact of the vaccination status, especially the role of the genotypic background of the respective seed virus, we analyzed NT-titers with respect to the vaccination status of the individual serum donors. The samples were grouped by immunization profile, resulting in three groups containing sera from persons vaccinated with Encepur^®^ (Encepur^®^; *n* = 12), FSME-IMMUN^®^ (FSME-IMMUN^®^; *n* = 11) or both vaccines (*MIX*; *n* = 10). Pairwise comparisons of the groups revealed significant differences (Kruskal–Wallis, *p* = 2.2 × 10^−16^), showing the highest NT-titers for the *MIX* group and the lowest NT-titers for the Encepur^®^ group (Figure 2a). The overall variances of NT-titers differed significantly between the groups (Fligner–Killeen, *p* = 0.04225), with lower intra-sample variances for sera from individuals immunized with both vaccines (Figure 2b).

Next, we projected and compared NT-titer phenotypes for the different TBEV-EU genotypes for each immunization profile group through time (Figure 2c). The individual phenogram plots provided a phylogenetically scaled view of mean NT-titers among the immunization profile groups, corrected for possible phylogenetic dependence of observed differences. This phylogenetic approach reconstructed the ancestral states of a given trait (mean NT-titer), based on the time-scaled phylogeny (Figure 1a), and allowed us to compare NT-titers among the three immunization profile groups in the phylogenetic context of each TBEV-EU genotype. The overall, pronounced, quantitative differences between the three profiles (Figure 2c) were consistent with the unscaled comparison (Figure 2a). The overall, pronounced, quantitative differences among the three profiles (Figure 2c) were consistent with the unscaled comparison (Figure 2a). Furthermore, the phenogram displayed a narrower spread of mean NT-titers in *MIX* and FSME-IMMUN^®^ group sera than in those from individuals immunized only with Encepur^®^. These observations were confirmed by Kruskal–Wallis tests that displayed no significant differences within the *MIX* (*p* = 0.74) and FSME-IMMUN^®^ groups (*p* = 0.066) and significant differences in NT-titers within the Encepur^®^ group (*p* = 6.3 × 10^−5^). *DZIF17_1133* and *K2* showed significantly lower NT-titers in the Encepur^®^ group, compared to the mean, while *Neudörfl* and *DZIF18_1750* displayed significantly higher titers (all *p* << 0.01).

### 3.4. Sequence Analysis of Possible Genetic Determinants for the NT-Titer Phenoytpes

Finally, we performed a sequence analysis of mutations in the polyprotein and individual mature proteins to investigate possible genetic determinants for the observed serological variabilities (Supplementary Figure S1). As the strain *K23* (seed virus for Encepur^®^) was not available in the study, we also compared it to the sequence of its progenitor strain *K2*. *K23* showed one substitution at N332K of the *E* protein, which was unique to this strain. Its progenitor, the strain *K2*, shared two substitutions, A363T and K416R, which were not present in the other used TBEV-EU strains. *Neudörfl* (seed virus for FSME-IMMUN^®^) showed one unique substitution in the *E* protein at V447I. No correlation among the amount of substitutions to *Neudörfl* and *K23* and the NT-titers was found. Furthermore, we identified four virus strains with identical *E* protein sequences and differing NT-titer results (*Schweizer_Isolat*, *DZIF15_33*, *DZIF15_55*, and *DZIF18_1133*). On the polyprotein level, all strains were unique, but we did not identify substitutions or sequence regions with accumulating substitutions that could be correlated with higher or lower NT-titers.

## 4. Discussion

Complex interactions between host and pathogen lead to a successful immune response and, thus, clearance of the pathogenic load. Vaccination aims to initiate the complicated host defense processes by the application of attenuated or inactivated pathogen structures. The goal is to protect the host from infection with a wild-type form of that pathogen. However, even small changes in the amino acid sequence or other virulence-determining regions may cause alterations in the pathogen’s epitope structure or replication capacity, which may result in reduced vaccine efficacy or even vaccination failures. Genotypic diversity may differ significantly for different pathogens and habitats, depending, e.g., on effective population size, gene flow, and migration patterns of pathogens and their vectors.

Thus, in addition to considering individual determinant factors of vaccination efficacy in the host, we need to study the impact of different genetic backgrounds and the potential genotypic diversity of the pathogens.

Tick-borne encephalitis is an emerging infection in Europe and Asia. Two vaccine brands are licensed in Europe. These brands are based on different seed–virus genotypes. Encepur^®^ contains the strain *K23*. FSME-Immun^®^ is based on the TBEV strain *Neudörfl*. Both seed–virus strains belong to the European subtype; however, they represent distinct phylogenetic subclades of TBEV-EU. In recent years, it has become more and more apparent that the increase and emergence of human TBE cases is accompanied by an increase in TBEV-EU diversity [18]. In the light of this development, it is unclear whether these two distinct vaccine strains differ in their capacity to induce neutralizing antibody responses to naturally occurring TBEV-EU strains.

In this study, we focused on the neutralization of 16 different TBE viruses by sera of vaccinated individuals, assessing the determinant factors in the host, the vaccine, and the TBEV genotypes. The goal was to investigate differences in neutralization activity among different viruses of the European subtype of TBEV. We sampled and sequenced additional isolates, emulating possible TBEV-EU encounters of vaccinated individuals traveling in high-risk regions in southern Germany and bordering countries. We further assessed the impact of different immunization profiles with different brands on the individual sera’s neutralization potentials.

We investigated the impact of different host factors. Previous studies showed that the administration of a prior yellow fever (YF) vaccination influences the immunization response against TBEV. While ELISA and hemagglutination inhibition tests often resulted in comparable titers, an overall negative effect on the formation of neutralizing antibodies could be identified [31,50]. While our data confirmed a very weak negative effect, we did not observe significant differences among the neutralization results from YF-vaccinated individuals compared to the non-YF-vaccinated group. Thus, the impact of a prior yellow fever vaccination was considered very low. This confirmed the view that the neutralization test is highly specific and seems, usually, not to react against other flavivirus or to booster a reaction against other flaviviruses. However, no information was available about whether the donors were vaccinated against YF prior to, after, or in-between the TBEV initial vaccinations.

Recent studies showed that females often mount a more robust humoral and cellular immune response than males [51,52]. Consistent with these prior studies, we observed a significant but weak positive correlation of NT-titers with the serum donor’s sex (polyserial correlation coefficient 0.121509184; *p* = 3.152979 × 10^−6^) In contrast to these data, no significant differences were observed related to the sex of the serum donors in our study. This may be explained by our lower sample size.

To analyze the age of the sample donors, we grouped them into three categories: *20–30* years, *31–40* years and *41–60* years. The groups differed significantly. The *31–40* group displayed the highest neutralization titers, while the *41–60* group showed the lowest titers. Similar data have been previously reported, leading to the proposal of shortening the booster intervals or to providing an extra priming dose for older persons [52,53,54].

We assumed that in the group of 31–40-year-old individuals, the percentage of those who still had a good immune response but had already obtained multiple shots was the highest. This also raised the possibility of mixed brands, which was another indicator for higher titers. Furthermore, it was previously shown that younger vaccinees produce higher antibody titers, but the quality of these antibodies is the same as those produced by elderly vaccinees with lower overall antibody titers [55].

In contrast to the studies cited above, our primary focus was on the potential impact of different genetic backgrounds and genotypic diversity of the pathogens and not on the study of host factors that determine vaccine efficacy. Thus, the composition of our study group was not designed to elucidate differences in sex and age of the sample donors. The presented descriptive statistics and tests of these host variables did not reveal compositional biases in our study group that affected our conclusions on the impact of the studied pathogenic factors.

There was a mild, but significant, positive relationship between the number of vaccination shots and the neutralization titers observed in the sample donors. This correlation was consistent with the global notion of the strong relationship between vaccine adoption and coverage in the population and TBEV immune efficacy [56,57,58]. A previous study showed that boostering, in a good immunological status may have had only a minor effect on antibody titers, in contrast to boostering in a status of low antibodies, when the increase of antibody formation was significant higher [59].

Overall, the influence of donor specific characteristics that might influence the outcome of the study results was low and the data were considered unbiased from these factors. The observed high inter-sera and low intra-serum variation indicated that the immune response of every donor was highly individual.

To better assess the extent to which these individual host factors contribute to vaccine effectiveness in light of the potential diversity of pathogen factors encountered in TBEV microfoci in the wild, we set out to challenge the 33 sera with a panel of TBEV-EU genotypes. The aim of this endeavor was to mimic the possible immune challenge of a vaccinated individual living and/or traveling in TBE hotspot areas in southern Germany or neighboring regions in Switzerland and Austria.

Given the close geographical proximity (Figure 1c), the selected local TBEVs from the European subtype displayed an astonishing diversity and, at first sight, an unexpected level of divergence in the performed in-depth phylogenetic analysis of the genomes. All of the other 14 TBEV-EU strains were grouped into 11 separate phylogenetic clades that were dated as having diverged more than 86 years ago, from either of the two seed viruses (*K23* and *Neudörfl*; Figure 1a). This observation is consistent with our previous description of genotypically divergent, locally restricted TBEV microfoci with limited-to-no gene flow and a low effective population size, due to migration barriers of the endemic vectors [18]. In this scenario, long-range dispersal dependent on random chance encounters with larger non-host targets, i.e., migratory vertebrates such as deer or humans.

Given this substantial level of divergence observed in the genomes, the most important and positive conclusion that can be drawn from our data is the overall observation that the sera from vaccinated individuals neutralized the overwhelming majority of the TBEV-EU genotypes in the panel. Neutralization titers could be detected in all but a single serum–virus combination (FSME-Immun^®^). This demonstrated strikingly that both vaccines are able to induce antibodies that are capable of neutralizing a wide variety of TBEV strains that are circulating in Central Europe (Figure 1b).

Phylogenetic comparative analysis of NT-titers along the dated tree of TBEV-EU strains indicated no significant phylogenetic signal and no significant differences between the identified clades, i.e., the phylogenetic distance from the respective seed virus was not a good predictor of neutralization efficiency (Figure 1b). Globally, in our dataset, there did not seem to be specific TBEV-EU lineages that were able to escape the antibodies raised by the available vaccines. Thus, our data were contrary to a generalizable genotypic factor among the TBEV-EU pathogens.

However, there were significant differences in the neutralization efficiency and the levels of inter-strain variance among the sera (Figure 2b). The individual sera differed substantially in their responses to certain genotypes. Passaging, storage, and handling conditions were mostly identical. Thus, there seemed to be individual host-pathogen interaction factors that affected neutralization efficiency. Even small numbers of amino acid substitutions may lead to conformational changes in the virus proteins and, hence, the viral antigens that, in turn, need to be recognized by the immune system built from a variable genetic makeup in the host. Therefore, we concluded that through this interaction the virus strain, which was used to initially prime the immune system and its phylogenetic distance to the encountered TBEV genotype, poses a host-specific influence on the neutralization titer levels.

Our further analyses showed that these inter-serum variations in neutralization efficiencies among multiple TBEV genotypes can be reduced and overall NT-titers increased by combing the two available vaccines. Analysis of the results according to the immunization profiles revealed that most virus strains showed significant differences in the three groups. Priming and/or boostering of the immune system with both *K23* and *Neudörfl* (*MIX* group) resulted in the highest NT-titers, while application of *K23* (Encepur^®^) resulted in the lowest NT-titers. The ranges of NT-titers in the three groups differed significantly. While the *MIX* and the FSME-IMMUN^®^ groups showed non-significant variations among the viruses, significant deviations were found in the Encepur^®^ group.

We identified strains that exhibit significantly higher or lower titers in the Encepur^®^ group, compared to the other two groups. The overall high variation among the neutralization results of the different strains within this group led to the assumption that the Encepur^®^ vaccine might lead to the production of antibodies that accept less variation in the target structures than does the FSME-IMMUN^®^ counterpart. This effect could be a result of the virus strain used in the vaccine. In contrast to strain *Neudörfl*, which in our taxon set first diverged from the last common ancestor of TBEV-EU strains, the divergence of *K23*, like many of the other strains, falls within the likely early diversification period of TBEV in Central Europe [18] and, thus, may represent a more derived state. The strain has one amino acid substitution, which was probably acquired during the cultivation process in vaccine seed–virus production and was found in any wild-type strains we studied. This single substitution in the *E* protein might lead to an altered antigen structure and, hence, to the production of highly specific antibodies with a narrower range. Similar results were reported by Beck et al. [29]. Nevertheless, all sera from Encepur^®^ vaccinees neutralized all the virus strains tested in the study.

Taking this data together, we concluded that different virus strains of a subtype are not neutralized alike, and even a single amino acid substitution might affect neutralization behavior. However, the study of Beck et al. differed from ours in study design. The used viruses were not naturally occurring strains, but were of hybrid origin. Furthermore, only sera from children were tested. It is known that children have a more active immune system and, therefore, may have reacted differently from adults [60]. The antigen dosage in both children’s vaccine formulations was only half of the formulation for adults. Therefore, the data of that study might be not directly transferred to the real situation. Therefore, we performed this pilot study to test sera from vaccinated adults with TBEV field isolates.

Starting from this observation, we set out to look for further substitutions that could be correlated with changes in NT-titers. However, consistent with the phylogenetic comparative analysis, our subsequent sequence analyses did not reveal direct links between neutralization titers and the nucleotide or amino acid sequence of the polyprotein or its components. As the major antigen, *Envelope protein* is the obvious target for such analyses. Strikingly, even TBEV-EU strains with identical *Envelope* gene sequences resulted in different neutralization titers. Salat et al. [61] showed that NS1 protein is present in the vaccines and that specific antigens can be found in vaccinated individuals. These antigens are said to be not neutralizing, but they may still alter the immune response and the production of neutralizing antibodies. Therefore, we investigated the *E* and the *NS1* protein sequences together. Combining the two proteins, all strains analyzed were unique, supporting the idea that stimulation with *NS1* antigen might influence the neutralizing antibody production. The strains *DZIF15_33* und *DZIF15_55* differ in only one amino acid substitution in the *NS1* protein. Our neutralization experiments resulted in substantially different titers for the two genotypes (Figure 1b), especially for the Encepur^®^ group. These findings demonstrate the importance of a thorough investigation of newly isolated TBEV strains and experimental testing to monitor the antigenic variation of the circulating strains to assess the neutralizing efficiency as one proxy for the immune efficacy of vaccines.

In general, the neutralization titers generated with samples from the Encepur^®^ group were lower and showed more variation than samples from the other two groups. Unfortunately, the vaccination seed strain *K23* was not available for this study. Thus, we could not test whether the single specific amino acid substitution in the *E* protein might be the basis for this observation. Although these results may lead to the assumption that the vaccination with Encepur^®^ results in different stimulation of antibody production than vaccination with FSME-IMMUN^®^, in light of previous data from a much larger study, in terms of the number of compared sera but with much lower numbers in tested TBEV genotypes [62], we should refrain from over-interpreting this aspect. This variation in the production of neutralizing antibodies also showed that vaccinees with Encepur^®^ neutralized all TBEV-EU strains used in this study, while one serum from the FSME-Immun^®^ group did not neutralize one TBEV-EU strain. Overall, the TBEV-EU strain *Neudörfl* produced significantly higher titers in our study. This might also lead to an overestimation of titers, especially in clinical studies, in comparison to other naturally circulating TBEV strains.

In our view, the more important takeaway from our data is the genotypic interaction effect of both the vaccine seed virus and the challenge virus. The use of an early diverging lineage (ancestral state) or a combination of multiple genotypes (or even genotypic lineages) seems to be clearly beneficial in raising effective antibodies.

To date, it has been unclear what influence these different antibody titer patterns may have on protection against infection. Although post-primary doses have not been specifically tested for TBE, the European Medicine Agency does not recommend drawing conclusions on the need for post-primary doses solely on antibody titers (Steffen, pers. information). In addition, mere serological tests do not seem to reflect real immune capacity and memory [63]. To date, there has been no evidence of a better field effectiveness of FSME-IMMUN^®^ vs. Encepur^®^. Possible interchangeability of the two vaccine brands has been reported by previous studies [62,64].

## 5. Conclusions

Our findings led us to conclude that both vaccines induce antibodies that are capable of neutralizing a wide range of TBEV strains of the European subtype. In our study, the number of vaccination shots did not strongly affect neutralization titers, but the used vaccine brand or a mix of brands did strongly affect neutralization titers. Vaccination profiles with broader or more basal genotypic coverage resulted in better and less-variable neutralization of diverse TBEV-EU strains. Even single amino acid substitutions in the different virus strains used in the study seemed to influence the outcome of the NT experiments, mainly in the groups vaccinated with only one vaccine brand. It seems that priming the immune system with two virus strains prepares well for addressing the different virus strains circulating in Central Europe. The administration of vaccine shots from different brands, therefore, might be an economically and serologically advisable way to address the immune system as broadly as possible and to reach higher neutralization titers and improved resilience.

## Figures and Tables

**Table 1:** Metadata of human sera samples.

Sera	Vaccination	Count	Age	Sex	YF Vaccination
HuSe002	FSME-IMMUN^®^	5	30–40	Male	Vaccinated
HuSe013	FSME-IMMUN^®^	3	20–30	Male	No vaccination
HuSe017	FSME-IMMUN^®^	3	40–60	Female	No vaccination
HuSe021	FSME-IMMUN^®^	3	20–30	Female	No vaccination
HuSe022	FSME-IMMUN^®^	4	40–60	Female	Vaccinated
HuSe026	FSME-IMMUN^®^	5	30–40	Male	Vaccinated
HuSe027	FSME-IMMUN^®^	6	30–40	Female	No vaccination
HuSe028	FSME-IMMUN^®^	6	30–40	Female	No vaccination
HuSe029	FSME-IMMUN^®^	5	20–30	Male	Vaccinated
HuSe035	FSME-IMMUN^®^	3	20–30	Female	No vaccination
HuSe034	FSME-IMMUN^®^	6	40–60	Female	Vaccinated
HuSe003	MIX	5	40–60	Male	Vaccinated
HuSe007	MIX	5	30–40	Female	Vaccinated
HuSe009	MIX	6	30–40	Female	Vaccinated
HuSe012	MIX	5	30–40	Female	No vaccination
HuSe015	MIX	5	20–30	Male	No vaccination
HuSe024	MIX	5	20–30	Female	No vaccination
HuSe025	MIX	5	40–60	Female	No vaccination
HuSe030	MIX	7	30–40	Male	No vaccination
HuSe031	MIX	3	30–40	Female	Vaccinated
HuSe038	MIX	4	20–30	Male	No vaccination
B1	Encepur^®^	4	20–30	Female	No vaccination
B5	Encepur^®^	3	20–30	Female	No vaccination
B8	Encepur^®^	5	20–30	Male	No vaccination
B9	Encepur^®^	4	20–30	Female	No vaccination
B11	Encepur^®^	5	20–30	Female	No vaccination
B12	Encepur^®^	4	20–30	Female	No vaccination
B13	Encepur^®^	6	20–30	Female	No vaccination
B14	Encepur^®^	4	20–30	Male	No vaccination
B18	Encepur^®^	6	20–30	Female	No vaccination
B20	Encepur^®^	4	20–30	Male	No vaccination
B23	Encepur^®^	4	20–30	Female	No vaccination
B24	Encepur^®^	5	40–60	Female	No vaccination

**Table 2:** TBEV-EU virus strains used in this study.

Strain ID	Acc.	Country	Region/City	Year of Isolation	Isolation Source	Passage History	Reference	Sequencing Method
K2 (Karlsruhe); K23	AM600965	Germany	Karlsruhe	1975	*Ixodes ricinus*	4. BMB 1. A549	[21] provided by FX Heinz, Vienna, Austria	Amplicon sequencing with Illumina TruSeq
Neudörfl	TEU27495	Austria	Neudoerfl	1971	*Ixodes ricinus*	Unknown	[22,23]	Amplicon sequencing with Illumina TruSeq
DZIF14 97		Austria	Aschau/Zillertal	2014	*Ixodes ricinus*	A549 Passage 01	Isolate of IMB	Amplicon sequencing with Illumina TruSeq
DZIF15 33		Germany/Austria	Eglsee/Kufstein	2015	*Ixodes ricinus*	A549 Passage 01	Isolate of IMB	Amplicon sequencing with Illumina TruSeq
DZIF15 569		Austria	Wald/Pitztal	2015	*Ixodes ricinus*	A549 Passage 01	Isolate of IMB	Amplicon sequencing with Illumina TruSeq
DZIF17 1044		Germany	Mühlau	2017	*Ixodes ricinus*	A549 Passage 01	Isolate of IMB	Amplicon sequencing with Illumina TruSeq
DZIF17 1989		Germany	Petting	2017	*Ixodes ricinus*	A549 Passage 01	Isolate of IMB	Amplicon sequencing with Illumina TruSeq
DZIF18 1133		Italy	Tres	2018	*Ixodes ricinus*	A549 Passage 01	Isolate of IMB	Amplicon sequencing with Illumina TruSeq
BaWa11 171	KX268728	Germany	Heselbach	2011	*Ixodes ricinus*	A549 Passage 01	[32]	Sanger sequencing
Schweizer_Isolat (40)		Switzerland	Schaffhausen	1972	*Ixodes ricinus*	1. BMB 1. A549	provided by Franz X. Heinz	Amplicon sequencing with Illumina TruSeq
BaWa13 203		Germany	Haselmühl	2013	*Ixodes ricinus*	A549 Passage 01	Isolate of IMB	Amplicon sequencing with Illumina TruSeq
DZIF17 2291		Germany	Nürnberg	2017	*Ixodes ricinus*	A549 Passage 01	Isolate of IMB	Amplicon sequencing with Illumina TruSeq
DZIF18 1750		Germany	Gleissenbach	2018	*Ixodes ricinus*	A549 Passage 01	Isolate of IMB	Amplicon sequencing with Illumina TruSeq
DZIF18 1648		Germany	Schnaittenbach	2018	*Ixodes ricinus*	A549 Passage 01	Isolate of IMB	Amplicon sequencing with Illumina TruSeq
CG/223_1990	KC835597	Slovakia	Zahorska Ves	1990	*Myodes glareolus*	5. BMB 3. Vero 1. A549	[33]	Sanger sequencing
DZIF15 55		Germany/Austria	Hechtsee	2015	*Ixodes ricinus*	A549 Passage 01	Isolate of IMB	Amplicon sequencing with Illumina TruSeq

Abbreviations: IMB = Bundeswehr Institute of Microbiology, BMB = baby mouse brain.

**Table 3:** Statistical analysis of demographic and vaccination related information.

	Neutralization Titers (Mean ± SD)/10 LOG2
Sex of the sample donors	
Female	*n* = 22 (66%) 3.79 ± 1.85
Male	*n* = 11 (33%) 4.17 ± 1.99
Wilcoxon Test	*p* = 0.063
Yellow fever vaccination	
YF+	*n* = 9 (27%) 3.8 ± 1.41
YF−	*n* = 24 (73%) 3.96 ± 2.06
Wilcoxon Test	*p* = 0.79
Number of Vaccinations (nV)	
3	*n* = 6 (18%) 3.83 ± 1.68
4	*n* = 8 (24%) 3.37 ± 1.93
5	*n* = 12 (36%) 4.19 ± 1.98
6	*n* = 6 (18%) 3.81 ± 1.73
7	*n* = 1 (4%) 6
Kendall’s rank correlation test	R = 0.109, *p* = 0.0018 **
Generalized linear model	nV-coefficient = 0.2393 *p* = 0.00212 **
Immunization profile	
Encepur	*n* = 12 (36.3%) 2.8 ± 1.43
FSME-IMMUN	*n* = 11 (33.3%) 3.8 ± 1.62
MIX	*n* = 10 (30.3%) 5.35 ± 1.74

** highlights significant differences.

## Data Availability

Not applicable.

## Supplementary Materials

The following supporting information can be downloaded at: https://www.mdpi.com/article/10.3390/vaccines11061044/s1. Table S1. Summary statistics of vaccination status. Table S2. Metadata of human sera samples. Excel sheet of Table 1. Table S3. TBEV-EU virus strains used in this study. Excel sheet of Table 2. Figure S1. Differences in the E-genes and NS1-Proteins on amino acid level. Substitutions are highlighted in color according to their biochemical characteristics.
