# Supplementary material for: Increased Vaccination Diversity Leads to Higher and Less-Variable Neutralization of TBE Viruses of the European Subtype
Source: Vaccines (Basel). 2023 May 31;11(6):1044. doi: 10.3390/vaccines11061044 (PMC10304915; doi:10.3390/vaccines11061044)
Supplement: Supplementary file 1 [file vaccines-11-01044-s001.zip › vaccines-2412326-supplementary.pdf]

Supplementary:

Table S1. Summary of Vaccination status.

Vaccination status	count		mean	median	sd	Se
Encepur	192		2.8	3	1.43	0.103
FSME-IM-MUN	176		3.80	4	1.62	0.122
MIX	160		5.35	6	1.74	0.137

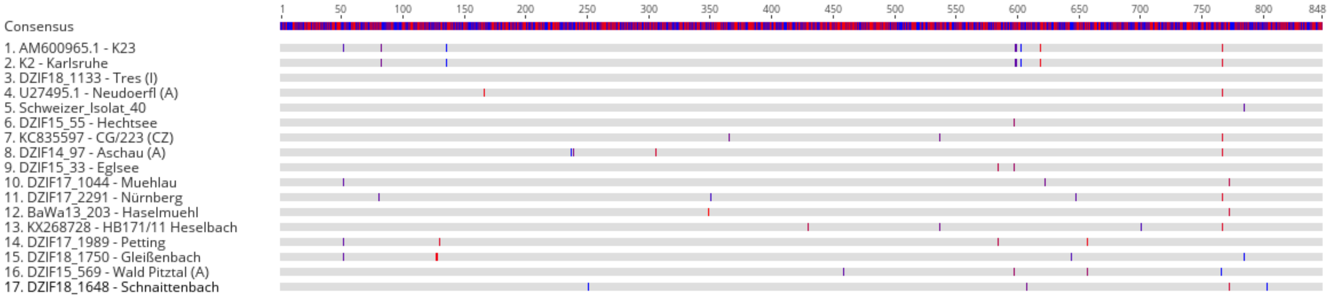

Figure S1. Differences in the E-genes and NS1-Proteins on amino acid level. Substitutions are highlighted in color according to their biochemical characteristics.
